# Quantification of HLA class I molecules on renal cell carcinoma using Edman degradation

**DOI:** 10.1186/1471-2490-11-1

**Published:** 2011-01-20

**Authors:** Juliane S Stickel, Natalie Stickel, Jörg Hennenlotter, Karin Klingel, Arnulf Stenzl, Hans-Georg Rammensee, Stefan Stevanović

**Affiliations:** 1 Department of Immunology, Institute for Cell Biology, University of Tübingen, Germany; 2 Clinic for Urology, University of Tübingen, Germany; 3 Department of Hematology Oncology, University of Freiburg, Germany; 4 Department of Molecular Pathology, University of Tübingen, Germany

## Abstract

**Background:**

Unimpaired HLA class I antigen presentation is a prerequisite for the recognition of tumor cells by cytotoxic T lymphocytes and thus essential for the success of anticancer immunotherapeutic concepts. Several approaches have been taken in the immunotherapy of metastatic renal cell carcinoma (RCC), however of limited success. HLA loss or down-regulation have often been reported and might interfere with immunotherapeutic approaches aimed at the recognition of HLA-presented peptides.

**Methods:**

We employed a quantitative method of molecular analysis for the comparison of HLA amounts on primary tumor, normal kidney and metastases of RCC, using Edman degradation. We analyzed a series of 47 RCC samples including corresponding renal parenchyma, local lymph node metastases and distant metastases.

**Results:**

Results of quantitative Edman degradation revealed significantly higher HLA yields on primary tumor and metastases compared to normal kidney tissue. This effect was shown not to result from infiltrating immune cells, since tumor-infiltrating lymphocytes had no influence on the overall HLA recovery from tumor tissue. Unexpectedly, we found a higher amount of HLA class I molecules on distant metastases compared to local lymph node metastases.

**Conclusion:**

Edman degradation allows the direct quantitative comparison of HLA class I protein expression by tumor or normal tissue and metastases of RCC patients. Our results raise hopes for improving the success and effectiveness of future immunotherapeutic concepts for metastatic RCC.

## Background

Metastatic renal cell carcinoma (RCC) remains a disease with fatal prognosis that is responsible for almost 100,000 deaths per year [1]. It is the most common malignant tumor in adult kidney, accounting for approximately 3% of human malignancies. Metastatic RCC is still a challenging tumor entity even though various strategies for chemotherapy have been developed in recent years [2,3]

The high immunogenicity of RCC has led to several immunotherapeutic concepts, starting with IFNα/IL-2 treatment [4] and advancing over numerous studies using cell-based vaccines to vaccination therapy with HLA-presented peptides derived from tumor-associated antigens [5-8]. Although some immunotherapeutic studies for metastatic RCC have found their way into the clinic [9-11], the clinical results of cancer immunotherapy are still below expectation.

Most immunotherapeutic concepts focus on HLA class I molecules, which are necessary for antigen presentation to cytotoxic T cells (CTLs). Even today the critical amount of HLA class I complexes on the cell surface that leads to T cell activation has still not been finally clarified - specifications vary between a few thousand and under ten [12,13]. Nevertheless, HLA class I altered expression on cancer cells still represents one of the most important mechanisms of tumor escape from immune response that eventually leads to accumulation of new variants with low immunogenicity and high capability for metastatic progression [14,15]. For this reason, the presentation of HLA class I antigens on tumor cell surface seems to be one of the main factors impairing the success and clinical outcome of peptide-based cancer vaccines aimed at increasing specific anti-tumor activity of CTLs [16]. Altered tumor expression of HLA class I is frequently observed in various types of malignancies and in some cases it has been associated with poor clinical prognosis [17-19]. In case of RCC, the downregulation or loss of HLA-class I has also been described [20,21], as well as overexpression of HLA class I on RCC compared to normal kidney tissue [22]. However, the quantification in these studies was only determined indirectly with immunohistochemistry or on mRNA level by gene expression analysis. While immunohistochemistry as a semi-quantitative method sheds a spotlight on only one layer of tissue, mRNA levels do not always correlate well with protein levels. Therefore, both methods might yield indirect information but only direct analysis of HLA expression on the protein level is capable of appropriate HLA class I quantification.

Thus, the aim of this study was to use a well-established quantitative molecular method [23] for the direct comparison of HLA class I molecules presented by RCC. For this goal we used Edman degradation as a tool to quantify HLA class I molecules isolated by immunoprecipitation. This method should easily reveal quantitative differences between HLA class I presentation on primary tumor, autologous kidney tissue and metastases of RCC and by doing so, predict the success of immunotherapeutic concepts for metastatic RCC.

## Methods

### Patients and tumor samples

Tissue samples from RCC patients were provided by the Clinic for Urology, University of Tübingen, Germany. We acquired tissue from primary RCC tumors, from corresponding normal kidney, and synchronous or metachronous metastases which occurred in contiguous lymph nodes, contralateral kidney or other viscera. Specimens were frozen in liquid nitrogen immediately after surgery and stored at -80°C until further use. In addition, a corresponding vital specimen of tumor tissue was collected as fresh tissue in RPMI and used by the T cell monitoring group, Department of Immunology, University of Tübingen, for collecting tumor infiltrating lymphocytes (TILs). This study has been approved by the local ethical review board.

### Peptide extraction

Frozen tumor, normal kidney and metastatic tissues were processed as described previously [24,25]. Briefly, peptides were isolated according to standard protocols by immunoprecipitation of HLA molecules from solid tissues using the HLA-A, -B, and -C specific antibody W6/32 coupled to CNBr-activated Sepharose (Roche Applied Science) followed by acid elution and subsequent ultrafiltration.

### Immunohistochemistry for the detection of MHC class I molecules

For antigen retrieval formaldehyde-fixed 5 μm paraffin sections from tumors were heated with 10 mmol/L citrate buffer (pH 6.0) for 5 minutes at 120°C followed by incubation with the mouse monoclonal anti-HLA-A/B/C antibody (clone W6/32, ATCC) for 1 h at room temperature. Subsequently, the sections were incubated with biotinylated anti-mouse-antibodies (Vector Laboratories, Inc., Burlingame, CA) for 30 minutes at room temperature, and a streptavidin-biotin-immunoperoxidase system (Vectastain Elite StreptABC, Vector Laboratories, Inc., Burlingame, CA) followed by DAB (Dako) and counterstained with hematoxylin. As a negative control, isotype-matched IgG were used in place of the primary antibody. Slides were viewed with a Zeiss Axioskop 40 microscope.

### Edman degradation

HLA yield was determined by Edman degradation using a pulsed-liquid protein sequencer Procise 494A (Applied Biosystems) equipped with a special C_18 _column for phenylthiohydantoin (PTH) amino acid analysis (Spheri-5 PTH 5 μm, 220 × 2.1 mm; PerkinElmer). Edman degradation chemistry efficiency is determined primarily by two chemical reactions. The first is a phenylisothiocyanate (PITC) coupling reaction to the N-terminus of a protein. PITC reacts with the free amino (NH2) group, resulting in an acid labile phenylthiocarbamyl derivative at the N-terminus of the protein. Subsequently, trifluoroacetic acid (TFA) is introduced to cleave the modified N-terminal amino acid from the protein. After further modification to a more stable phenylthiohydantoin (PTH) derivative, the derivatized amino acid is chromatographed. The PTH amino acid is identified by its unique retention time in the chromatogram. This process is repeated iteratively for each subsequent terminal amino acid of the protein.

With the known N-terminal sequence of the HLA class I molecule it is possible to calculate the yield of HLA in each sample by determining the amount of PTH amino acids over the sequencing cycles. For this study we sequenced and analyzed the first seven amino acids of the HLA class I α-chain (GSHSMRY) which are conserved among all human class I molecules. Due to background effects of the abundant amino acids serine and glycine, the contaminating sequence NIVMTQS from Ig kappa chains, and varying yields of the somewhat unstable arginine [23], we focused in particular on the amount of histidine in the third cycle after subtracting the background value of the preceding cycle. Recombinant HLA class I monomers served to verify the reliability of our method. Samples of tumor, normal tissue and metastases acquired from the same patient were always sequenced directly in series, thus avoiding the influence of any day-to-day variation in this nonetheless quite robust analytical method.

### Normalization and statistical analysis

Results of quantitative Edman degradation were normalized according to the HLA yield from 1 g of tissue. Statistical analysis for significant differences in HLA yields was performed using the unpaired Student's t-test (Graph Pad Prism 5 Software).

## Results

The general outline of the study was to establish a method for direct HLA quantification from lysates of RCC. We performed immunoprecipitation of HLA class I molecules using affinity chromatography and determined the yield of eluted HLA class I molecules by quantitative Edman degradation. Tissues analyzed within this study are shown in Table 1. Quantification was carried out for a total of 47 RCC caucasian patients. From 5 RCC patients we could acquire triplet tissue samples from tumor, corresponding kidney tissue and metastases. In addition, 19 autologous pairs of tumor and normal renal tissue could be analyzed in this study. From one patient we obtained only normal tissue and a distant metastasis from contralateral kidney. In addition, 17 single primary tumors, 4 single metastases and 1 single normal tissue were analyzed. All examined tissues were processed with identical methods and the same protein sequencer.

**Table 1:** Tissue samples and amounts of immunopurified HLA class I

Tissue specimen	Tumor (T) Normal renal tissue (NK) Metastasis (M)	Type	Mass (g)	TILs	Edman results (pmol)	Average HLA amount (pmol/g)
RCC399	NK T M (lymph node)	ccRCC	2.4 13.8 1.0	yes	60 1000 40	25.0 72.5 40.0
RCC377	NK T M (lymph node)	ccRCC	4.2 12.7 4.5	n.d	500 6000 3500	119.0 472.4 777.8
RCC364	NK T M (lymph node)	ccRCC	1.2 5.8 0.4	yes	10 1000 20	8.3 172.4 50.0
Rcc343	NK T M (liver)	ccRCC	3.2 2.1 3.2	n.d	20 200 1400	6.3 95.2 437.5
RCC70	NK T M (lymph node)	ccRCC	4.4 8.3 2.6	n.d.	300 3200 200	68.2 385.5 76.9
RCC307	NK T	pRCC	2.7 1.6	n.d.	200 400	74.1 250.0
RCC211	NK T	pRCC	0.4 20.7	n.d.	10 2100	25.0 101.4
RCC193	NK T	ccRCC	2.4 4.7	n.d.	500 850	208.3 180.9
RCC131	NK T	ccRCC	6.5 11.5	n.d.	400 300	61.5 26.1
RCC121	NK T	ccRCC	6.2 10.4	no	500 460	44.2 80.6
RCC119	NK T	ccRCC	8.9 7.8	yes	440 560	49.4 71.8
RCC110	NK T	chRCC	13.0 7.0	yes	1700 3900	130.8 557.1
RCC100	NK T	ccRCC	12.6 8.0	no	1400 1200	111.1 150.0
RCC99	NK T	ccRCC	12.4 7.7	no	1500 1300	121.0 168.8
RCC81	NK T	chRCC	10.0 18.0	no	1400 1800	100.0 140.0
RCC76	NK T	ccRCC	6.2 12.8	n.d.	1200 2600	193.5 20.3
RCC71	NK T	ccRCC	9.0 7.0	n.d.	10 3000	1.1 428.6
RCC58	NK T	ccRCC	7.6 7.2	no	2000 2400	263.2 333.3
RCC57	NK T	n.d.	6.8 8.7	yes	1900 2500	279.4 287.4
RCC53	NK T	n.d.	6.9 6.2	n.d.	1300 1900	188.4 306.5
RCC52	NK T	ccRCC	7.4 7.0	n.d.	100 2000	13.5 285.7
RCC51	NK T	chRCC	7.0 7.8	n.d.	2100 2000	300.0 256.4
RCC49	NK T	n.d.	7.0 8.8	n.d.	1300 2900	185.7 329.5
RCC46	NK T	n.d.	7.6 9.3	n.d.	400 1500	52.6 161.3
RCC173	NK M (contralateral k.)	ccRCC	5,0 5,1	n.d.	40 240	8.0 47.1
RCC249	T	chRCC	2.6	n.d.	80	30.8
RCC231	T	pRCC	6.3	n.d.	380	60.3
RCC226	T	ccRCC	6.8	n.d.	250	36.8
RCC195	T	ccRCC	60.0	n.d.	50	0.8
RCC130	T	ccRCC	6.0	no	1100	183.3
RCC125	T	ccRCC	15.5, 8.1	n.d.	400	25.8
RCC116	T	ccRCC	15.8	yes	2380	150.6
RCC115	T	ccRCC	26.0	yes	12900	496.2
RCC113	T	ccRCC	10.1	n.d.	200	19.8
RCC108	T	pRCC	30.0	yes	1500	50.0
RCC103	T	chRCC	10.0	yes	4400	440.0
RCC101	T	ccRCC	7.5	yes	800	106.7
RCC98	T	ccRCC	21.1	n.d.	2100	99.5
RCC90	T	ccRCC	20.0	n.d.	10200	510.0
RCC75	T	pRCC	16.0	n.d.	2200	137.5
RCC73	T	ccRCC	10.0	n.d.	1300	130.0
RCC68	T	ccRCC	20.0	yes	2500	125.0
RCC395	M (lymph node)	ccRCC	1.4	n.d.	50	35.7
RCC333	M (pancreas)	ccRCC	10.9	yes	3000	275.2
RCC328	M (contralateral k.)	ccRCC	1.5	n.d.	300	200.0
RCC112	M (adrenal gland)	ccRCC	2.5	n.d.	700	280.0
RCC126	NK	ccRCC	8.9	yes	200	22.5

Total and normalized HLA yields of all tissue samples analyzed in this study. Weight, histological subtype and information about present TILs are indicated for each examined RCC (tumor, normal tissue and metastases). HLA typing was carried out by the Institute for Clinical and Experimental Transfusion Medicine and histologic classification was performed by the Department of Pathology, University of Tübingen, Germany. T, tumor; NK, normal kidney; M, metastasis; n.d., not determined; ccRCC, clear cell renal cell carcinoma; pRCC, papillary renal call carcinoma; chRCC, chromophilic renal cell carcinoma; no, no TILs detected; yes, TILs detected.

HLA yield appeared to be very heterogeneous with total HLA class I amounts ranging from 10 to 12900 pmol respectively from 0.8 to 777.8 pmol/g for normalized HLA yield (Table 1).

### Overall quantitative comparison

**Figure 1:**
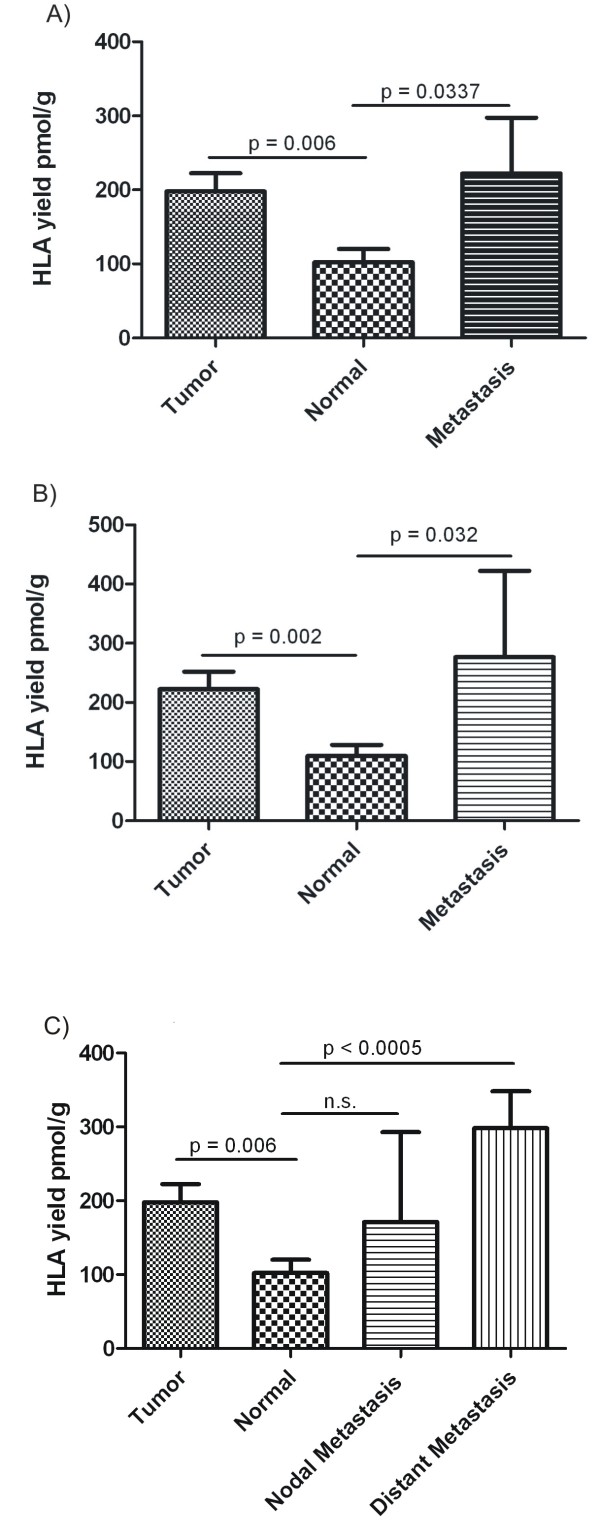
**HLA yields from different tissues were normalized to 1 g of tissue**. The mean value of the normalized HLA yield of the respective tissue type is given plus standard error of the mean (SEM). Statistical significance of the differences between tissues is reflected in p values determined by the Student`s t-test and indicated in the histogram. Amounts of immunopurified HLA class I compared between: A) Primary tumors (Tumor), normal tissues (Normal) and metastases of all analyzed tissue samples (overall comparison). B) Primary tumor, corresponding normal tissue and metastases obtained from the same patient (autologous comparison). C) Primary tumor, normal tissue, lymph node metastases and distant metastases of all analyzed tissue samples.

Table 1 shows the overall HLA yield determined by Edman degradation and the normalized HLA yield in pmol/g. For statistical analyses we used unpaired Student's t-tests to identify significant differences in HLA yield from different tissues. Overall comparison between tumor, normal tissue and metastases of all 47 analyzed RCC samples is shown in Figure 1A. The mean amount of HLA class I was 197.8 ± 24.6 pmol/g recovered from tumor tissue (n = 40), 102.3 ± 17.9 pmol/g from normal kidney tissue (n = 26) and 222.0 ± 75.4 pmol/g from metastases (n = 10). Significant differences in HLA yield were observed for the comparison of tumor and normal tissue (p = 0.0062) as well as for metastases and normal tissue (p = 0.0337). The overall comparison clearly showed an increased yield of HLA class I from tumor and metastases of RCC compared to normal kidney tissue. There was also a slight increase of HLA yield on metastases compared to tumor tissue but below the level of statistical significance (p = 0.69).

Looking at the differences between tumor, normal tissue and metastases in the autologous setting (*i.e*., samples acquired from the same individual) to achieve a direct comparison of the HLA yield (Figure 1B), we determined the mean amount of HLA class I at 222.2 ± 29.6 pmol/g on tumor tissue (n = 24), 109.6 ± 18.6 pmol/g on normal kidney tissue (n = 24) and 276.4 ± 145.7 pmol/g on metastases (n = 5). Again, statistically significant differences in HLA yield were detected for the comparison between tumor and normal tissue (p = 0.0023) as well as for the comparison between metastases and normal tissue (p = 0.0329). Comparison between tumor and metastases showed again a slight but non-significant increase in HLA yield from metastases, due to low sample numbers. This direct autologous comparison confirmed the results of the overall comparison, demonstrating an increased amount of HLA class I on tumor and metastases of RCC compared to normal kidney tissue.

### Differences between local lymph node metastases and distant metastases of RCC

In order to distinguish between HLA quantities recovered from lymph node metastases and distant metastases (derived from pancreas, liver, adrenal gland and controlateral kidney), we compared tumor and normal kidney tissue to lymph node metastases and distant metastases, separately (Figure 1C). The mean amount of HLA class I detected was 171.3 ± 121.5 pmol/g from local lymph node metastases (n = 6) and 298.2 ± 49.9 pmol/g on distant metastases (n = 4). Higher HLA yield from distant metastases compared to normal tissue showed a statistical significance (p = 0.0005), whereas the comparison between normal tissue and lymph node metastases could not reach a level of significance. Thus HLA presentation on distant metastases of RCCs appears to be higher than on lymph node metastases.

### Impact of tumor infiltrating lymphocytes on the HLA yield of RCC

**Figure 2:**
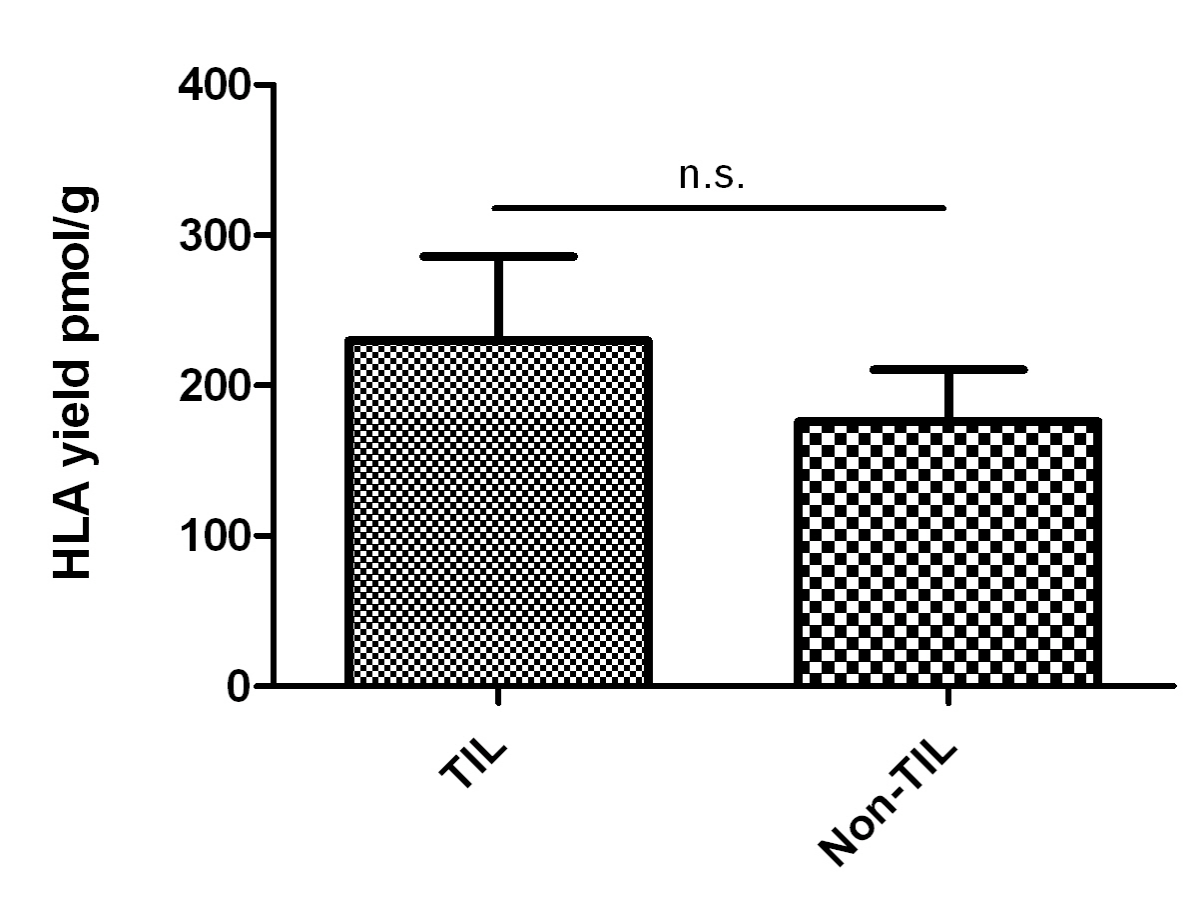
**Comparison TILs vs. no TILs**. Amounts of immunopurified HLA class I compared between primary tumors containing TILs and tumor tissue without TILs. Yields were normalized to 1 g of tissue. The mean value of the normalized HLA yield of the respective tissue type is given plus standard error of the mean (SEM). Statistical significance of the differences between tissues is reflected in p values determined by the Student`s t-test and indicated in the histogram.

In order to rule out the possibility that tumor infiltrating lymphocytes (TILs) have a strong impact on quantitative HLA analysis by Edman degradation, we compared primary RCCs from which TILs could be cultured with tumors from which no TILs could be isolated and cultured (Figure 2). The mean amount of HLA class I was 230.0 ± 55.9 pmol/g recovered from RCC with TILs (n = 11) compared to 176.0 ± 34.6 pmol/g from tumors without TILs (n = 6). This comparison shows that the HLA amount in tumors containing TILs is slightly higher than in tumors without TILs. However, statistical analysis showed no significance; the p value of 0.51 suggests a nearly identical HLA yield on both tumor groups.

### Immunohistochemical analysis of HLA class I expression

**Figure 3:**
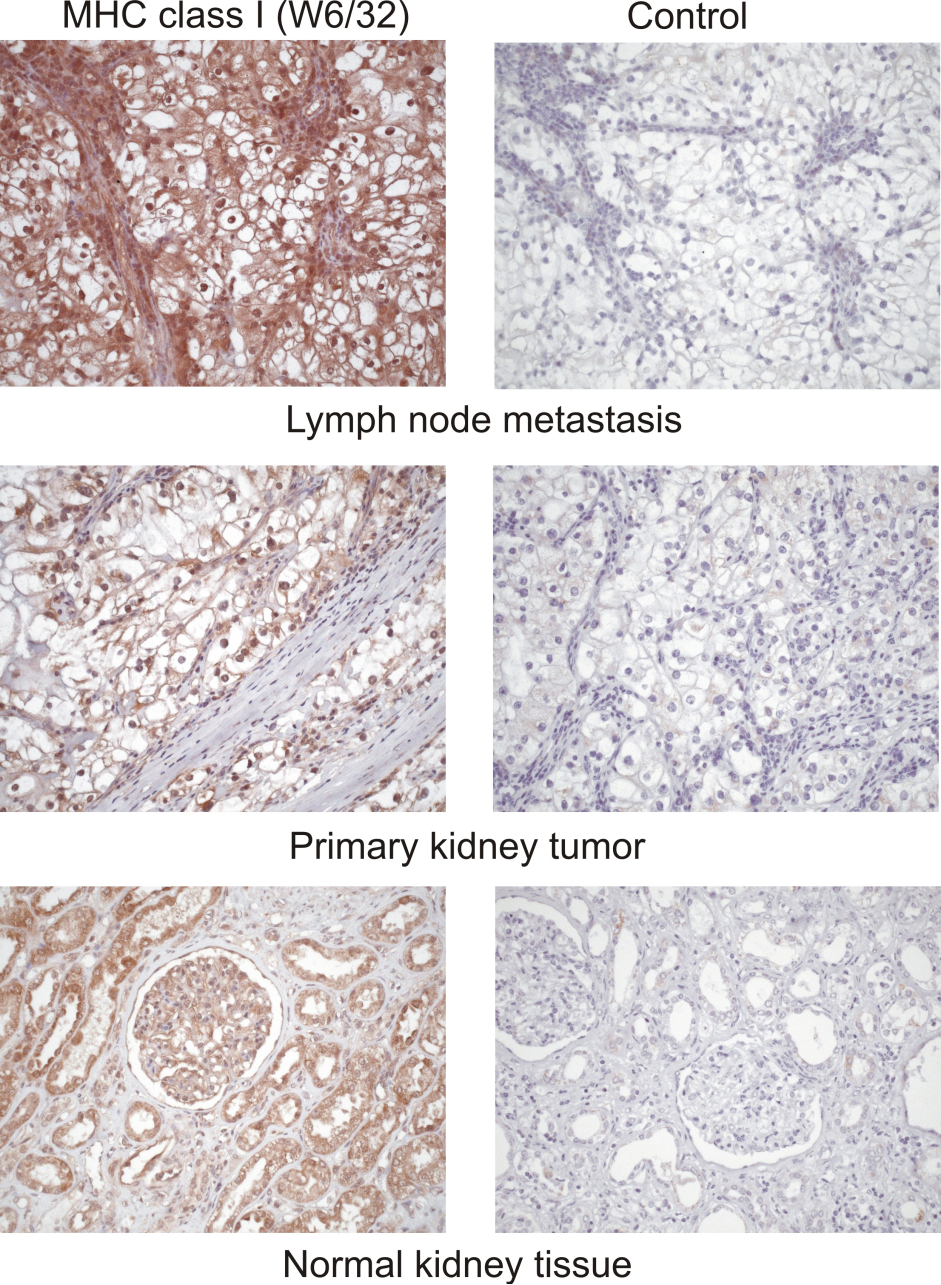
**Immunohistochemical staining of RCC377 tissues**. Staining with the HLA class I specific antibody W6/32 demonstrates a higher expression of class I in tumor vs. benign kidney tissue. Expression in metastatic tissue is still higher. Although antigen presenting cells (APC) and endothelial cells contribute to HLA class I expression, tumor cells localized within this lymph node metastasis are also expressing class I at a high level.

We performed immunohistochemistry as a complementary method to visualize HLA class I expression in different tissues of our patients. Figure 3 shows exemplarily our findings in RCC377, revealing an abundant HLA class I expression in a lymph node metastasis as well as a stronger expression in primary tumor cells in comparison to normal kidney tissue. Within the lymph node metastasis, epithelial cells and immune cells contribute to HLA class I expression but tumor cells are also intensely stained.

## Discussion

### Edman degradation for quantitative HLA analysis

Although other techniques have in the meantime surpassed Edman chemistry in sensitivity, cost, and use for routine protein characterization, automated Edman degradation remains the most effective tool for obtaining N-terminal amino acid sequence information, and represents a reliable quantitative technique in its routine application [26]. Therefore we chose Edman degradation with the intention of establishing a method for direct quantitative analysis of HLA molecules immunopurified from RCC. To our knowledge this is the first study comparing HLA yield on tumor, normal tissue and metastases of RCC using direct quantification. Previous studies used indirect methods, such as immunofluorescence, or analysed the HLA class I expression on mRNA level [20,27,28].

Considering intrinsic effects of automated Edman degradation chemistry, such as inefficiencies in the coupling and cleavage reactions as well as different recoveries of PTH amino acids, we sequenced and analyzed the first seven amino acids of the HLA class I α-chain (GSHSMRY), focusing especially on quantitation of histidine in the third cycle. Raw values were neither corrected for repetitive yields (for the instruments operating in most laboratories, a 92-94% overall repetitive yield is considered acceptable [26]) nor for initial yields or PTH amino acid recoveries [23]. The first monitoring measurements with known yields of recombinant HLA class I monomers evinced histidine in sequence position 3 as most reliable parameter for the overall HLA amount of the analyzed sample. Other authors reported of large errors in the calculated amount of peptides when several different amino acids were used for repetitive and initial yield calculations [26,29].

The aim of this study was not to establish a method for determining the absolute amount of HLA class I molecules on the cell surface, but to obtain relative amounts that are comparable between different types of tissues. A study of the Edman Sequencing Research Group (ESRG) showed that the relative amounts comparing two different samples were highly accurate, the average error being 6.8% [26].

Thus Edman degradation proved to be an appropriate method for direct quantitative comparison of HLA class I molecules immunopurified from tumor, normal tissue and metastases of RCC patients. We have to emphasize, however, that rather large samples are required with respect to the sensitivity of Edman sequencers. Our study included tissues of far more than 2 g in most cases; the need for a big sample size prevented the analysis of larger cohorts.

### Results of quantitative HLA analysis

The presentation of HLA class I antigens on tumor cells represents a major factor in determining the success and clinical outcome of immunotherapeutic concepts aimed at increasing specific anti-tumor activity of CTLs. In order to predict the success of immunotherapeutic approaches for metastatic RCC, it was decided to compare HLA class I presentation on primary tumor, autologous normal kidney tissue and metastases of renal cell carcinoma. We performed this comparison on different levels, including local and distant metastases. To our knowledge this is the first study determining HLA class I yield directly on the protein level from RCCs and metastatic tissue.

The HLA yield was found in most cases to be dependent on the tissue mass (Table 1). Low amounts of HLA class I recovered from bulky tumors with a mass up to 60 g are presumably due to necrotic decay or stromal invasion of these tumors *in vivo*.

The overall comparison of all 47 analyzed tissue samples (Figure 1) as well as the direct comparison of 24 autologous pairs of tumor and normal tissue (Figure 2) showed a significantly higher yield of HLA class I from tumor than normal tissue.

It has to be mentioned that tumors represent heterogeneous tissues which contain different subsets of cells. Although RCC is expected to be more homogenous than, for example, colon carcinoma, the tumors analyzed in this study might harbor endothelial cells and - most important - immune cells that could distort HLA quantification because of their high HLA expression. To demonstrate that a greater HLA yield from the tumors was indeed due to a higher HLA class I amount on tumor cells and not to tumor infiltrating lymphocytes (TILs), which could be found in 70% of all renal cell carcinomas (Table 1 and C. Gouttefangeas, unpublished), we compared primary RCCs from which TILs could be cultured with tumors from which no TILs could be isolated (Figure 2). Although we observed a tendency of TIL-containing tumors to yield slightly more HLA class I molecules than the TIL-free samples, the differences were not statistically significant (p = 0.51) and thus much smaller than the differences between tumor and normal tissue. From these data there seems to be no major impact of TILs on the results of quantitative HLA analysis in RCC tumors. On the other hand, we cannot rule out completely that endothelial cells or antigen presenting cells [20,21] contribute to the enhanced recovery of HLA molecules from tumor tissue and in particular from lymph node metastases. Immunohistochemical staining of tissues, as exemplarily shown in RCC377, confirms abundant expression of HLA class I on the surface of tumor cells but also endothelial and immune cells. Figure 3 gives an impression of HLA density on the different subsets of cell types. As outlined above, however, immunhistochemistry as a semi-quantitative method gives information on one layer of tissue only.

Recent reports have described a total or partial loss of HLA class I for nearly all types of tumors [18,30]. HLA class I downregulation or complete loss was observed in 10 - 50% of melanomas, breast, lung, colorectal, cervix and prostate cancers [19]. In case of RCC, downregulation and loss of HLA class I has also been described: The amount of HLA negative tumors was determined to be up to 37.8% and was correlated with lower 5-year survival rates [20]. On the other hand, higher HLA expression in RCC compared to normal kidney tissue has been reported as well [22,31]. The individual analysis of our study indicates a slight HLA downregulation on tumor tissue compared to normal tissue for only 5 of 24 tumors and normal tissue pairs (20.8%) (Table 1), whereas for most of the tumor tissues considerably higher HLA class I amounts were quantified. There was no significant difference in HLA presentation on different subtypes of RCCs investigated in this study.

Since T-cell based immunotherapy is not expected to clear bulky tumor masses, many clinical applications focus on the stage of minimal residual disease. To combat micrometastases remaining after dissection of the primary tumor, the metastases must express certain HLA levels and present tumor-associated peptides. In a recent report we confirmed that tumor-associated HLA ligands are indeed shared between primary tumors and metastases [32]. Here, we performed a quantitative comparison of HLA class I molecules immunopurified from metastases, primary tumors, and normal tissues (Figures 1A, B) and detected significantly higher HLA amounts on metastatic tissue compared to normal kidney tissue. HLA class I yield from metastatic tissue was slightly higher than from tumor tissue, but beyond the level of significance. Contrary to the expectation that lymph node metastases, due to their higher amount of immune cells, should contain more HLA class I molecules, the comparison between lymph node metastases and distant metastases (Figure 1C) revealed a significantly higher amount of HLA on distant metastases than on normal tissue and lymph node metastases. These findings are in contrast to reports of HLA down-regulation or loss even stronger in metastases than in primary tumors [33]. However, HLA loss rates vary between different tumor entities [34] and there are only few reports on HLA expression in distant metastases of RCC. Numerous reports have correlated tumor progression with downregulation or complete loss of HLA [16,21,35], for example in metastatic melanoma a high HLA class I amount (on metastases) correlated with regression whereas a low HLA amount correlated with progression under therapy [16]. These facts and our data hold promise for the success of immunotherapeutic strategies also against metastatic RCC.

## Conclusion

The aim of our study was to establish a direct method for quantitative analysis of HLA class I molecules immunopurified from tumor, normal tissue, and metastases of RCC. Edman degradation appears to be a feasible, effective and reliable method for performing quantitative direct comparisons of samples of sufficient size. Our results may contradict other reports, but they do provide an impulse for improving the effectiveness of current and future immunotherapeutic concepts for metastatic RCC. Due to the limited amount of samples analyzed and the heterogeneity of the individual HLA yields our results cannot be generalized at present.

## Abbreviations

CNBr: Cyanogen bromide; CTL: Cytotoxic T lymphocyte; ESRG: Edman Sequencing Research Group; HLA: Human leukocyte antigen; IFN Interferon; IL: Interleukin; PITC: Phenylisothiocyanate; PTH: Phenylthiohydantoin; RCC: Renal cell carcinoma; ccRCC: Clear cell renal cell carcinoma; chRCC: Chromophobe renal cell carcinoma; pRCC: Papillary renal cell carcinoma; RNA: Ribonucleic acid; TFA: Trifluoroacetic acid; TILs: Tumor infiltrating lymphocytes

## Competing interests

The authors declare that they have no competing interests.

## Authors' contributions

JS carried out the immunoprecipitation and Edman analysis and drafted the manuscript. NS carried out the statistical analysis. JH and AS provided the tumor, normal and metastasis tissue. KK performed immunohistochemical stainings. SS and HGR participated in the design of the study and its coordination. All authors read and approved the final manuscript.

## Pre-publication history

The pre-publication history for this paper can be accessed here:

http://www.biomedcentral.com/1471-2490/11/1/prepub

## References

[B1] Vogelzang NJ Stadler WM Kidney cancer Lancet 1998 352 9141 1691 1696 10.1016/S0140-6736(98)01041-1 9853456

[B2] Ravaud A Wallerand H Culine S Bernhard JC Fergelot P Bensalah K Patard JJ Update on the medical treatment of metastatic renal cell carcinoma Eur Urol 2008 54 2 315 325 10.1016/j.eururo.2008.04.056 18485581

[B3] Tamaskar I Pili R Update on novel agents in renal cell carcinoma Expert Rev Anticancer Ther 2009 9 12 1817 1827 10.1586/era.09.157 19954293

[B4] Coppin C Porzsolt F Awa A Kumpf J Coldman A Wilt T Immunotherapy for advanced renal cell cancer Cochrane Database Syst Rev 2005 1 CD001425 15674877 10.1002/14651858.CD001425.pub2

[B5] Schendel DJ Dendritic cell vaccine strategies for renal cell carcinoma Expert Opin Biol Ther 2007 7 2 221 232 10.1517/14712598.7.2.221 17250460

[B6] Uemura H Fujimoto K Tanaka M Yoshikawa M Hirao Y Uejima S Yoshikawa K Itoh K A phase I trial of vaccination of CA9-derived peptides for HLA-A24-positive patients with cytokine-refractory metastatic renal cell carcinoma Clin Cancer Res 2006 12 6 1768 1775 10.1158/1078-0432.CCR-05-2253 16551861

[B7] Suekane S Nishitani M Noguchi M Komohara Y Kokubu T Naitoh M Honma S Yamada A Itoh K Matsuoka K Phase I trial of personalized peptide vaccination for cytokine-refractory metastatic renal cell carcinoma patients Cancer Sci 2007 98 12 1965 1968 10.1111/j.1349-7006.2007.00631.x 17919310 PMC11159169

[B8] Van Poppel H Joniau S Van Gool SW Vaccine therapy in patients with renal cell carcinoma Eur Urol 2009 55 6 1333 1342 10.1016/j.eururo.2009.01.043 19201522

[B9] Brossart P Dendritic cells in vaccination therapies of malignant diseases Transfus Apher Sci 2002 27 2 183 186 10.1016/S1473-0502(02)00041-1 12350054

[B10] Kubler H Vieweg J Vaccines in renal cell carcinoma Semin Oncol 2006 33 5 614 624 10.1053/j.seminoncol.2006.06.011 17045091

[B11] Wierecky J Muller MR Wirths S Halder-Oehler E Dorfel D Schmidt SM Hantschel M Brugger W Schroder S Horger MS Immunologic and clinical responses after vaccinations with peptide-pulsed dendritic cells in metastatic renal cancer patients Cancer Res 2006 66 11 5910 5918 10.1158/0008-5472.CAN-05-3905 16740731

[B12] Garcia KC Degano M Pease LR Huang M Peterson PA Teyton L Wilson IA Structural basis of plasticity in T cell receptor recognition of a self peptide-MHC antigen Science 1998 279 5354 1166 1172 10.1126/science.279.5354.1166 9469799

[B13] Kageyama S Tsomides TJ Sykulev Y Eisen HN Variations in the number of peptide-MHC class I complexes required to activate cytotoxic T cell responses J Immunol 1995 154 2 567 576 7814868

[B14] Chang CC Ferrone S Immune selective pressure and HLA class I antigen defects in malignant lesions Cancer Immunol Immunother 2007 56 2 227 236 10.1007/s00262-006-0183-1 16783578 PMC11030175

[B15] Garrido F Algarra I MHC antigens and tumor escape from immune surveillance Adv Cancer Res 2001 83 117 158 full_text 11665717 10.1016/s0065-230x(01)83005-0

[B16] Aptsiauri N Carretero R Garcia-Lora A Real LM Cabrera T Garrido F Regressing and progressing metastatic lesions: resistance to immunotherapy is predetermined by irreversible HLA class I antigen alterations Cancer Immunol Immunother 2008 57 11 1727 1733 10.1007/s00262-008-0532-3 18491093 PMC11030993

[B17] Aptsiauri N Cabrera T Garcia-Lora A Lopez-Nevot MA Ruiz-Cabello F Garrido F MHC class I antigens and immune surveillance in transformed cells Int Rev Cytol 2007 256 139 189 10.1016/S0074-7696(07)56005-5 17241907

[B18] Rolland P Deen S Scott I Durrant L Spendlove I Human leukocyte antigen class I antigen expression is an independent prognostic factor in ovarian cancer Clin Cancer Res 2007 13 12 3591 3596 10.1158/1078-0432.CCR-06-2087 17575223

[B19] Seliger B Cabrera T Garrido F Ferrone S HLA class I antigen abnormalities and immune escape by malignant cells Semin Cancer Biol 2002 12 1 3 13 10.1006/scbi.2001.0404 11926409

[B20] Kitamura H Honma I Torigoe T Asanuma H Sato N Tsukamoto T Down-regulation of HLA class I antigen is an independent prognostic factor for clear cell renal cell carcinoma J Urol 2007 177 4 1269 1272 discussion 1272 10.1016/j.juro.2006.11.082 17382705

[B21] Kitamura H Torigoe T Honma I Sato E Asanuma H Hirohashi Y Sato N Tsukamoto T Effect of human leukocyte antigen class I expression of tumor cells on outcome of intravesical instillation of bacillus calmette-guerin immunotherapy for bladder cancer Clin Cancer Res 2006 12 15 4641 4644 10.1158/1078-0432.CCR-06-0595 16899613

[B22] Saenz-Lopez P Gouttefangeas C Hennenlotter J Concha A Maleno I Ruiz-Cabello F Cozar JM Tallada M Stenzl A Rammensee HG Higher HLA class I expression in renal cell carcinoma than in autologous normal tissue Tissue Antigens 75 2 110 118 10.1111/j.1399-0039.2009.01409.x 19912575

[B23] Stevanovic S Jung G Multiple sequence analysis: pool sequencing of synthetic and natural peptide libraries Anal Biochem 1993 212 1 212 220 10.1006/abio.1993.1314 8368496

[B24] Falk K Rotzschke O Stevanovic S Jung G Rammensee HG Allele-specific motifs revealed by sequencing of self-peptides eluted from MHC molecules Nature 1991 351 6324 290 296 10.1038/351290a0 1709722

[B25] Weinzierl AO Maurer D Altenberend F Schneiderhan-Marra N Klingel K Schoor O Wernet D Joos T Rammensee HG Stevanovic S A cryptic vascular endothelial growth factor T-cell epitope: identification and characterization by mass spectrometry and T-cell assays Cancer Res 2008 68 7 2447 2454 10.1158/0008-5472.CAN-07-2540 18381453

[B26] Brune DC Hampton B Kobayashi R Leone JW Linse KD Pohl J Thoma RS Denslow ND ABRF ESRG 2006 study: Edman sequencing as a method for polypeptide quantitation J Biomol Tech 2007 18 5 306 320 18166674 PMC2392991

[B27] Atkins D Ferrone S Schmahl GE Storkel S Seliger B Down-regulation of HLA class I antigen processing molecules: an immune escape mechanism of renal cell carcinoma? J Urol 2004 171 2 Pt 1 885 889 10.1097/01.ju.0000094807.95420.fe 14713847

[B28] Ibrahim EC Allory Y Commo F Gattegno B Callard P Paul P Altered pattern of major histocompatibility complex expression in renal carcinoma: tumor-specific expression of the nonclassical human leukocyte antigen-G molecule is restricted to clear cell carcinoma while up-regulation of other major histocompatibility complex antigens is primarily distributed in all subtypes of renal carcinoma Am J Pathol 2003 162 2 501 508 12547708 10.1016/S0002-9440(10)63844-8 PMC1851152

[B29] Smithies O Gibson D Fanning EM Goodfliesh RM Gilman JG Ballantyne DL Quantitative procedures for use with the Edman-Begg sequenator. Partial sequences of two unusual immunoglobulin light chains, Rzf and Sac Biochemistry 1971 10 26 4912 4921 10.1021/bi00802a013 5134536

[B30] Watson NF Ramage JM Madjd Z Spendlove I Ellis IO Scholefield JH Durrant LG Immunosurveillance is active in colorectal cancer as downregulation but not complete loss of MHC class I expression correlates with a poor prognosis Int J Cancer 2006 118 1 6 10 10.1002/ijc.21303 16003753

[B31] Romero JM Aptsiauri N Vazquez F Cozar JM Canton J Cabrera T Tallada M Garrido F Ruiz-Cabello F Analysis of the expression of HLA class I, proinflammatory cytokines and chemokines in primary tumors from patients with localized and metastatic renal cell carcinoma Tissue Antigens 2006 68 4 303 310 10.1111/j.1399-0039.2006.00673.x 17026465

[B32] Stickel JS Weinzierl AO Hillen N Drews O Schuler MM Hennenlotter J Wernet D Muller CA Stenzl A Rammensee HG HLA ligand profiles of primary renal cell carcinoma maintained in metastases Cancer Immunol Immunother 2009 58 9 1407 1417 10.1007/s00262-008-0655-6 19184600 PMC11031011

[B33] Lopez-Nevot MA Esteban F Ferron A Gutierrez J Oliva MR Romero C Huelin C Ruiz-Cabello F Garrido F HLA class I gene expression on human primary tumours and autologous metastases: demonstration of selective losses of HLA antigens on colorectal, gastric and laryngeal carcinomas Br J Cancer 1989 59 2 221 226 10.1038/bjc.1989.45 2649129 PMC2246991

[B34] Blades RA Keating PJ McWilliam LJ George NJ Stern PL Loss of HLA class I expression in prostate cancer: implications for immunotherapy Urology 1995 46 5 681 686 discussion 686-687 10.1016/S0090-4295(99)80301-X 7495121

[B35] Bukur J Malenica B Huber C Seliger B Altered expression of nonclassical HLA class Ib antigens in human renal cell carcinoma and its association with impaired immune response Hum Immunol 2003 64 11 1081 1092 10.1016/j.humimm.2003.08.350 14602239

